# Nano-Assemblies of Modified Cyclodextrins and Their Complexes with Guest Molecules: Incorporation in Nanostructured Membranes and Amphiphile Nanoarchitectonics Design

**DOI:** 10.3390/nano4030741

**Published:** 2014-08-20

**Authors:** Leïla Zerkoune, Angelina Angelova, Sylviane Lesieur

**Affiliations:** CNRS UMR 8612 Institut Galien Paris-Sud, Paris-Sud 11 University, 92290 Châtenay-Malabry, France; E-Mails: leila.zerkoune@u-psud.fr (L.Z.); sylviane.lesieur@u-psud.fr (S.L.)

**Keywords:** amphiphilic cyclodextrins, self-assembly, cyclodextrin nanoassemblies, mixed supramolecular cyclodextrin materials, nanoparticles, nanoencapsulation, gene and drug delivery

## Abstract

A variety of cyclodextrin-based molecular structures, with substitutions of either primary or secondary faces of the natural oligosaccharide macrocycles of α-, β-, or γ-cyclodextrins, have been designed towards innovative applications of self-assembled cyclodextrin nanomaterials. Amphiphilic cyclodextrins have been obtained by chemical or enzymatic modifications of their macrocycles using phospholipidyl, peptidolipidyl, cholesteryl, and oligo(ethylene oxide) anchors as well as variable numbers of grafted hydrophobic hydrocarbon or fluorinated chains. These novel compounds may self-assemble in an aqueous medium into different types of supramolecular nanoassemblies (vesicles, micelles, nanorods, nanospheres, and other kinds of nanoparticles and liquid crystalline structures). This review discusses the supramolecular nanoarchitectures, which can be formed by amphiphilic cyclodextrin derivatives in mixtures with other molecules (phospholipids, surfactants, and olygonucleotides). Biomedical applications are foreseen for nanoencapsulation of drug molecules in the hydrophobic interchain volumes and nanocavities of the amphiphilic cyclodextrins (serving as drug carriers or pharmaceutical excipients), anticancer phototherapy, gene delivery, as well as for protection of instable active ingredients through inclusion complexation in nanostructured media.

## 1. Introduction

Self-assembled nanomaterials of amphiphilic molecules have attracted considerable recent interest for diverse biomedical applications including the development of drug delivery systems and stimuli-responsive nanodevices [1,2,3,4,5,6,7,8,9,10]. Nano-objects (nanoparticles, liposomes, nanocapsules, nanospheres) with interesting properties can be fabricated from lipids, polymers, and/or amphiphilic cyclodextrins among other building blocks. Attention has been paid also to the synthesis of hybrid amphiphilic molecules, which can convey new structural features, bioactive and/or photoactive functionalities, capacity for transport through biomembranes, and a possibility for nanocarrier detection in physiological medium. The investigations of the structure-property and structure-activity relationships are thus of fundamental importance.

Grafting of hydrophobic moieties on the primary or the secondary faces of natural cyclodextrins (cyclic oligosaccharides forming nanocavity structures) confers them an amphiphilic character [11,12,13,14,15,16,17,18,19,20,21]. Synthesis of various modified cyclodextrins has been undertaken because such amphiphilic compounds may self-organize in aqueous phase to form supramolecular assemblies as drug carriers of high stability upon dilution [22,23,24,25,26,27,28]. The combination between the ability of the cyclodextrin derivatives to form colloidal suspensions of nano-sized dispersed particles and their capacity to form inclusion complexes with hydrophobic drugs has distinguished these systems as pretty valuable for drug encapsulation [29,30,31,32,33,34,35,36,37,38,39]. An important feature for the drug delivery field comes from the fact that the majority of amphiphilic cyclodextrins are considered to be non-hemolytic and non-cytotoxic [40,41,42,43,44,45,46,47,48]. Mixed amphiphilic nanostructures of phospholipids and modified cyclodextrin have been of special interest because these systems may display high affinity for interaction with biological membranes [49,50,51,52,53,54,55,56,57].

The first part of this review briefly presents the different classes of amphiphilic cyclodextrins. The second part describes the supramolecular assemblies of amphiphilic cyclodextrins alone or in self-assembled mixtures with other molecules of therapeutic significance. Different potential applications of the nanoassemblies formed by amphiphilic cyclodextrins upon interaction with biomolecules and other amphiphiles are outlined in the last part of the review.

## 2. Amphiphilic Cyclodextrins

The concept of amphiphilic cyclodextrins is based on modulation of the hydrophobic/hydrophilic balance of their construction as well as of their self-assembly properties through grafting of single or multiple substituents on the primary, secondary or both faces of native cyclodextrins [11,12,13,14,15,16,17,18,19,20,21,22,23,24,25,26,27,28,29,30,31,32,33,34,35,36,37,38,39,40,41,42,43,44,45,46,47,48,49,50,51,52,53,54,55,56,57,58,59,60,61,62,63,64,65,66,67]. Cyclodextrin modifications can be performed by enzymatic pathways [17,20,22] or by chemical conjugation reactions using amino, amido, thio, ester, ether or fluoro bonds [11,12,42,61,62,63,64,65,66,67]. The versatility of this approach has led to the synthesis of a huge number of amphiphilic cyclodextrin systems [68,69,70,71,72,73,74,75,76,77,78,79,80,81,82].

Three major classes of amphiphilic cyclodextrins can be distinguished as a function of the modifications of the cyclodextrin rims:

(i) Cyclodextrins with hydrophobic anchors on the primary face: “Medusa-like” molecules;
(ii) Cyclodextrins with hydrophobic anchors on the secondary face: “Skirt-shaped” molecules;
(iii) Cyclodextrins with hydrophobic or hydrophilic anchors on the both faces: “Bouquet-like” molecules.

**Figure 1:**
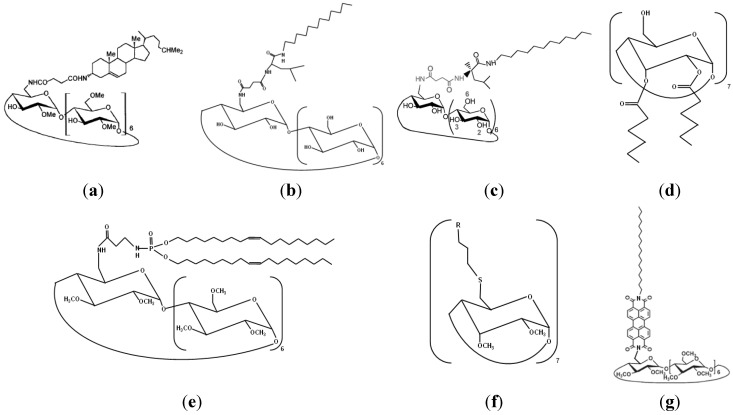
Amphiphilic cyclodextrins obtained by modifications of the macrocycle: (**a**) cholesteryl-cyclodextrin; (**b**) peptidolipidyl-cyclodextrin; (**c**) monolauryl-cyclodextrin; (**d**) hexanoyl-cyclodextrin; (**e**) phospholipidyl-cyclodextrin; (**f**) fluorinated-cyclodextin (βC_6_F_13_: R=C_6_F_13_); and (**g**) octadecylperylene-cyclodextrin. The chemical structures in (b), (d), (e), and (f) depict the compounds studied in refs. [12,23,41,42] respectively. (**a**) Reprinted with permission from [39]. Copyright (2000) American Chemical Society. (**c**) reprinted with permission from [54]. Copyright (2013) American Chemical Society. (**g**) reprinted with permission from [38]. Copyright (2010) American Chemical Society. Methylation of the secondary cyclodextin faces in (**a**), (**e**), (**f**), (**g**) yields permethylated derivatives.

The first two categories of compounds may be subdivided also into monosubstituted and persubstituted amphiphilic cyclodextrins. Figure 1 shows a few examples of modified cyclodextrins as representative derivatives in this review on self-assembled amphiphilic nanoarchitectures.

### 2.1. Cyclodextrins Modified by Large Moieties on the Primary Face

#### 2.1.1. Monosubstituted Amphiphilic Cyclodextrins

Amphiphilic cyclodextrins, monosubstituted on the primary face, have been obtained by anchoring of single hydrophobic moieties such as cholesterol, alkyl chains of variable lengths, fluorinated chains, or more complex synthetic peptidolipidyl and phospholipidyl substituents (see for instance Figure 1b,c).

One of the purposes of the design and preparation of these amphiphilic derivatives has been the development of nanocarriers with an affinity for insertion into model lipid bilayers and natural biological membranes. Another purpose has been the formation of colloidal supramolecular aggregates, in which the cyclodextrin cavities may serve as drug transporter units.

In this context, Auzély-Velty *et al* [11,39] reported the grafting of cholesteryl moieties, through spacers of various lengths, on the primary hydroxyl groups of the β-cyclodextrin molecule (which was permethylated on its secondary face). The original amphiphilic compounds have shown a capacity to incorporate into phospholipid bilayer systems. This property may be exploited for enhancement of the cellular membrane permeability of drug-loaded cyclodextrin nanocarriers.

#### 2.1.2. Polysubstituted Amphiphilic Cyclodextrins

Di- and polysubstituted amphiphilic cyclodextrins have been obtained by grafting of aliphatic chains or cholesteryl moieties on the primary hydroxyl groups of the cyclodextrin rim via amine, sulphur or sulfoxide linkers [13,14,15,16,53,54]. Studies of the interfacial properties of these compounds have shown that they may form stable monomolecular layers at the air-water interface [13,14,15,16]. For alkyl chain lengths longer than eight carbon atoms (C_8_), the monolayer transfer from the air/water interface to a solid support has yielded Langmuir-Blodgett multilayer films [15]. The recognition properties of the cyclodextrin nanocavities have been demonstrated by the Langmuir-Blodgett technique using azobenzene isomers as guest molecules dissolved in the aqueous subphase beneath the amphiphilic monolayers [14,15]. High monolayer integrity has been observed with a dicholesteryl-substituted cyclodextrin [16]. However, this compound, depending on its molar fraction in binary systems, has shown a tendency for two-dimensional phase segregation in mixed monolayers with phospholipids, for instance those of dipalmitoyl-L-α-phosphatidylcholine (DPPC) [16].

### 2.2. Cyclodextrins Modified on the Secondary Face

Amphiphilic cyclodextrins of a “skirt-shape” type have been prepared by esterification of the hydroxyl groups on the secondary face of native cyclodextrins using acyl donors with variable chain lengths (C_4_–C_16_). This esterification has been achieved through chemical or enzymatic pathways [17,18,19,20,21,22,23,24,25,26]. The obtained cyclodextrin derivatives (see for instance Figure 1d [23]) have self-organized into various supramolecular assemblies upon nanoprecipitation in solvents [21,22,23]. Their studies as potential drug carriers have established that the morphological (spherical, rod-like, irregular shape) features of the nanoparticles are crucial in determining the drug transport, the loading capacity of the nanocarriers for drugs, the *in vitro* release profiles, and the associated pharmacological parameters [24,25,26]. For decanoate-β-cyclodextrin esters, the inner nanoparticle organization has been shown to be of a multilamellar onion-like type [17]. Such multilamellar organization may be expected to provide enhanced nanoencapsulation for guest poor-soluble small drug molecules (e.g., diazepam, indomethacin, bifonazole, clotrimazole). Steric stabilization of the cyclodextrin nanocarriers against aggregation has been achieved by adding of surfactant shells on the particles (e.g., Pluronic F68, Span 85, Montane 80) in some cases [21].

### 2.3. Cyclodextrins Modified on Both Faces

Figure 1e–g shows examples of cyclodextrin structures modified on both faces, the secondary faces being substituted by methyl groups. In the work of Gervaise *et al* [12], two long phospholipidyl chains were grafted through an amine function on the cyclodextrin ring (Figure 1e). The physicochemical characterization of the obtained cyclodextrin derivatives has established their tensioactive properties, provoking spontaneous self-assembly in water and formation of nanoparticles, which might serve as drug nanocarriers. However, fusion of the nanoparticles into larger aggregates (>350 nm) upon storage has indicated the need of their steric stabilization against aggregation by means of additional polar substitutions.

“Bouquet-like” cyclodextrin molecules have been produced by grafting of hydrophobic (aliphatic chains) and hydrophilic substituents on the primary and secondary faces, respectively, of the cyclodextrin macrocycles in different configurations yielding libraries of new compounds [27,28,29,30,31,32]. Depending on the balance between the hydrophobic and hydrophilic anchors, supramolecular aggregates have been spontaneously assembled in a broad concentration range in aqueous medium. The introduction of hydrophilic oligo(ethylene glycol) chains onto the secondary face of the cyclodextrin ring has enhanced the aqueous solubility of the novel amphiphilic derivatives [30]. In addition, it has improved the long circulating properties of the nanoassemblies [58]. Based on structural measurements, some of these chemically modified β-cyclodextrins have been suggested to form core-shell types of micellar aggregates of diverse shapes [27]. On the other hand, cyclodextrin compounds modified on both faces have been developed with the purpose to use them as artificial transmembrane receptors [28]. They have been embedded in either liposome membranes or in giant unilamellar vesicles of phospholipids. “Bouquet-like” cyclodextrin derivatives, substituted on both sides of the ring, have been studied as porphirin-encapsulating carrier-sensitizer system for photodynamic anticancer therapy [29,30,32].

## 3. Supramolecular Assemblies of Amphiphilic Cyclodextrins

**Figure 2:**
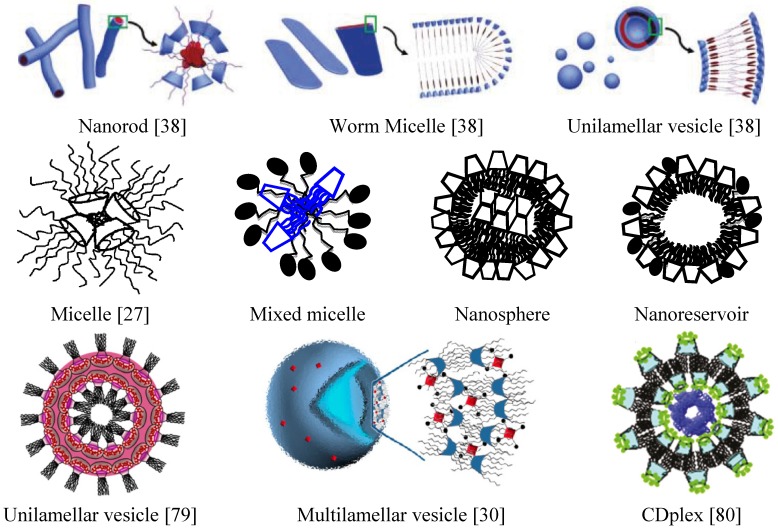
Schematic presentations of nanoarchitectures and nanoparticles involving amphiphilic cyclodextrins [27,30,38,79,80]. Reprinted with permission from [27]. Copyright (2004) American Chemical Society. Reprinted with permission from [30]. Copyright (2013) American Chemical Society. Reprinted with permission from [38]. Copyright (2010) American Chemical Society. Reprinted with permission from [79]. Copyright (2014) American Chemical Society. Reprinted with permission from [80]. Copyright (2011) American Chemical Society.

Amphiphilic cyclodextrins may self-assemble into a variety of nanoobjects [18,19,20,21,29,30,31,32,33,34,35,36,37,38,39,40,41,42,43,44,45,46,47,48] depending on the chemical nature, geometrical arrangement and lipophilicity of their grafted moieties (Figure 2 and Table 1). The aggregation process upon self-assembly may depend also on the concentration of the studied amphiphiles, the solvent medium, and temperature [38]. Nanoprecipitation has been the first method proposed for nanospheres preparation with high yield [19,23,37].

**Table 1:** Nanosystems formed by self-assembly of amphiphilic cyclodextrins.

Amphiphilic cyclodextrins	Organized systems	Refs.
2,3-diacyl-*O*-β-cyclodextrin	Nanocapsules	[21]
Heptakis(2-ω-amino-*O*-oligo(ethylene oxide)-6-hexylthio)-β-CD (SC6CDNH2)	Nanoparticles	[29]
Heptakis(2-*O*-oligo(ethylene oxide)-6-hexylthio)-β-CD (SC6OH)	Nanoparticles	[30]
2,3-di-*O*-hexanoyl cyclomaltoheptaose (βCD-C6)	Nanocapsules	[31]
SC6NH2@SC6Dns	Nanoparticles	[32]
2,6-di-*O*-methyl-β-CD	Micelles	[33]
βCD-C6	Nanospheres/Nanocapsules	[34]
βCD-C6/6-*N*-CAPRO-β-CD	Nanospheres	[35]
βCD-C6/6-*N*-MYRISTO-β-CD	Nanocapsules	[36]
βCD-C6/βCD-C12/ βCD-C14	Nanospheres	[37]
N-Octadecylperylene 3,4:9,10 tetra-caboxylic-3,4-permethyl-β-cyclodextrin-9,10-imide	Nanorods, Micelles,Vesicles	[38]
6^I^-(Cholest-5-en-3α-ylamido)succinylamido-6^I^-deoxy-*per*(2,6-di-*O*-methyl)cyclomaltoheptaose	Micelles	[39]
Mono [6-(2-aminohexylamino)-6-deoxy]-β –cyclodextrin	Nanorods, vesicles	[40]
Hexakis[6-deoxy-6-(3-perfluorohexylpropanethio)-2,3-di-*O*-methyl]-α-cyclodextrin (α-C6F13) or β-C6F13	Nanospheres	[41]
*N*-dodecyl-*N*α-(6^I^-amidosuccinyl-6^I^-deoxy-2^I^,3^I^-di-*O*-methyl-hexakis-(2^II^-V^II^,3^II^-V^II^,6^II^-V^II^tri- *O*-methyl)-cyclomaltoheptaose)-*L*-leucine	Micelles/Colloidal aggregates	[42]
Folate-polycationic amphiphilic CD	Nanocomplexes with DNA	[43]
Polycationic amphiphilic CD	Nanocomplexes with siRNA	[44]
Polycationic glyco-amphiphilic CDs	Nanocomplexes with DNA	[45]
Polycationic amphiphilic CD	Nanocomplexes with DNA	[45,46]
Poly-6-cationic amphiphilic CD	Nanocomplexes with DNA	[46]
Cationic amphiphilic β-cyclodextrins (hydrophobic *n*-alkylthio chains (C16) at the primary hydroxyl face and hydrophilic ω-amino-oligo(ethylene glycol) units at the secondary face)	Bilayer vesicles	[47]
SC8CDcysteamine (lipophilic group on the secondary face)	Nanocomplexes with siRNA	[48]
SC8CDcysteamine (lipophilicgroup on the primary face)	Nanocomplexes with siRNA	[48]

In the following, we discuss some interesting examples of organized amphiphilic cyclodextrin systems.

### 3.1. Colloidal Systems Involving Amphiphilic Cyclodextrins

**Figure 3:**
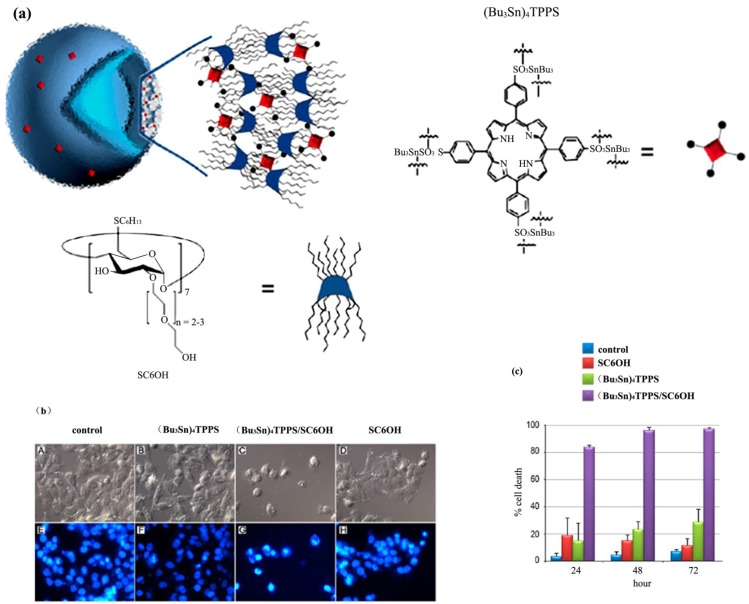
Supramolecular assemblies of amphiphilic cyclodextrins studied *in vitro* as potential nanotherapeutics [30]. (**a**) Chemical composition of the nanoassemblies of SC6OH (heptakis(2-*O*-oligo(ethylene oxide)-6-hexylthio)-β-CD) and (Bu_3_Sn)_4_TPPS (*meso*-tetra(4-sulfonatophenyl)porphine tributyltin(IV) derivative) formed in aqueous medium; (**b**) nuclear morphology of A375 melanoma cells treated with (Bu_3_Sn)_4_TPPS/SC6OH nanoassemblies; (**c**) cytotoxic effects of (Bu_3_Sn)_4_TPPS/SC6OH nanoassemblies on the A375 human melanoma cells viability. Reprinted with permission from [30]. Copyright 2013, American Chemical Society.

Cyclodextrin/porphyrin (SC6OH/(Bu_3_Sn)_4_TPPS) nanoassemblies with improved stability in aqueous medium and anticancer properties have been reported by Mazzaglia *et al.* [30]. The balance between the hydrophobic and hydrophilic chains grafted on both sides of the cyclodextrin macrocycle (Figure 3) has been crucial for modulation of the lypophilicity of the generated complexes, their cellular internalization efficiency, and cytotoxicity. Intracellular delivery studies of the SC6OH/(Bu_3_Sn)_4_TPPS nanoparticles in A375 human melanoma cells have established that the porphyrin species may be transported by the SC6OH nanocarriers in order to enter into the nucleus of the cells and concentrate in the nucleoli. The morphology of the melanoma cells has changed and the cell proliferation has been blocked at low concentration of the nanoassemblies, whereas high concentrations have induced the apoptosis of the A375 melanoma cells.

**Figure 4:**
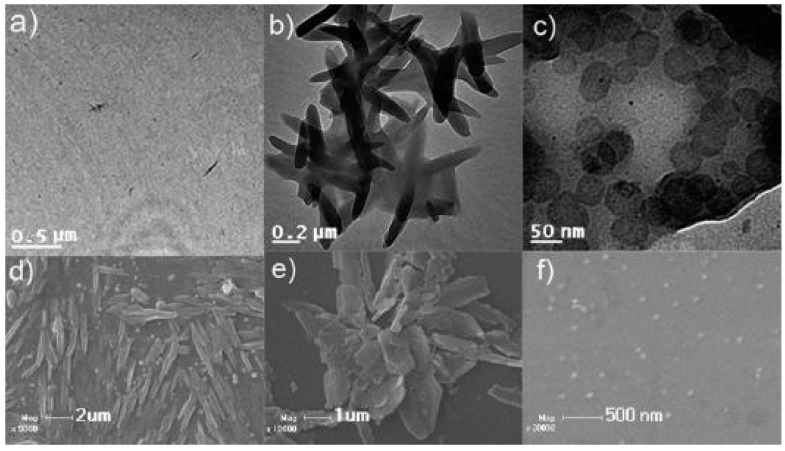
TEM (**a**–**c**) and SEM (**d**–**f**) images showing the solvent-dependent morphologies of supramolecular assemblies formed by an amphiphilic octadecylperylene-cyclodextrin derivative in mixed solvents with increasing polarity. From left to right: methanol, 4:6 water/methanol mixture, and 9:1 water/methanol mixture [38]. Reprinted with permission from [38]. Copyright 2010, American Chemical Society.

The effect of the solvent polarity on the self-assembled morphologies and aggregation behavior of an amphiphilic octadecylperylene-cyclodextrin has been demonstrated by Jiang *et al.* [38]. The authors have synthesized an asymmetric water-insoluble cyclodextrin-perylene conjugate (see the chemical structure shown in Figure 1g, bottom right panel). Depending on the solvent medium (binary water/methanol mixtures of varying volume ratios), the morphologies of the self-assembled cyclodextrin architectures have evolved from nanorods (formed in pure methanol) to floppy micelles (generated in a 4:6 water/methanol mixture) and spherical vesicles (stable in a 9:1 water/methanol mixture) (see the shapes in Figure 2, top line). The microscopic examination of the dry aggregate morphologies by transmission electron microscopy (TEM) and scanning electron microscopy (SEM) has confirmed the suggested solution-phase self-assembly pathways (Figure 4). Applications of such nanoassemblies have been foreseen in fluorescence sensory materials, photoinduced electron transfer systems, photoresponsive materials, and organic electronic devices [38]. A morphological transformation from nanorods to vesicles has been reported also for a mono[6-deoxy-N-*n*-hexylamino-(N′-1-anthraquinone)]-β-cyclodextrin derivative as a function of the composition of the aqueous phase [40].

**Figure 5:**
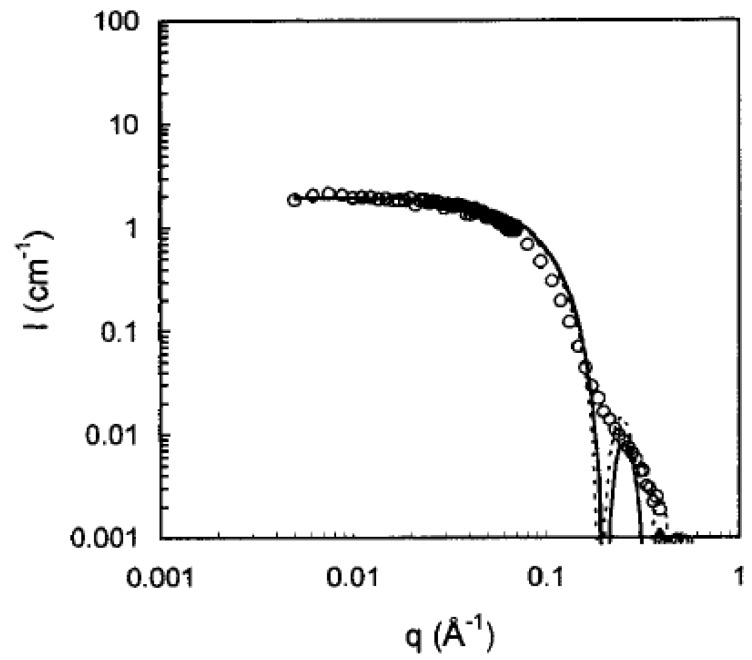
SANS spectrum acquired with monodispersed Chol-DIMEB micelles (10^−^^2^ M) assembled in D_2_O solution at 25 °C. The solid line represents the calculated model curve for non-interacting two-shell spherical micelles. The dotted line represents the calculated model curve for independent homogeneous spherical micelles [39]. Reprinted with permission from [39]. Copyright 2000, American Chemical Society.

Amphiphilic cholesteryl cyclodextrins, such as Chol-DIMEB, have been shown to self-aggregate into core-shell particles (Figure 5) in the form of micelles that are of interest for drug delivery applications [39]. Because of the small sizes (~5 nm) of the micellar structures formed by the monosubstituted cyclodextrins, molecular dynamics simulations and small-angle neutron scattering (SANS) (rather than electron microscopy) have been preferably employed to determine the aggregation number, the dimensions and shape of the resulting supramolecular aggregates [33,39]. The cyclodextrin nanocavities in the micellar assemblies have been established to be exposed toward the aqueous phase. In this manner, they possessed sufficient flexibility for capturing of guest drug molecules [33]. Similarly to non-ionic detergent micelles, the micellar assemblies of amphiphilic cyclodextrins have displayed enhanced solubilization capacity for poor soluble molecules [39].

Chiral peptidolipidyl-cyclodextrins have been designed based on a hybrid concept [42] with the purpose of preparing bioactive nanocarriers adapted for interaction with biological interfaces and membranes. The obtained peptidolipidyl-cyclodextrin derivative *N*-dodecyl-*N_α_*-(6^I^-amidosuccinyl-6^I^-deoxy-2^I^,3^I^-di-*O*-methyl-hexakis-(2^II^-^VII^,3^II^^–^^VII^,6^II^^–^^VII^-tri-*O*-methyl)-cyclomaltoheptaose)-*L*-leucine has been shown to spontaneously organize in supramolecular architectures upon dispersion in buffer medium. Its propensity to form colloidal aggregates and solubilize hydrophobic dye molecules has been studied by optical density (OD) measurements, quasi-elastic light scattering (QELS), NMR and UV-Visible spectroscopy [42]. The obtained nanoparticles have been established to exceed in size the dimensions of the globular micelles formed by cholesteryl cyclodextrins [39]. The observed aggregation behavior has been found to be reversible.

Cyclodextrin nanospheres of 2,3-di-*O*-hexanoyl cyclomaltooctaose (γ-CDC6) have been prepared by the nanoprecipitation method and their physical stability upon addition of solubilizing surfactant *n*-octyl-β-D-glucopyranoside (OG) was examined by Lemos-Senna *et al.* [55]. It has been established that the non-ionic detergent, at a critical ratio reached upon continuous addition of OG solution to the γ-CDC6 nanospheres, begins to disrupt the spherical organization of the initial particles (nanospheres). Evidence for the formation of mixed γ-CDC6-OG micelles has been obtained under solubilizing conditions (*i.e.*, at elevated OG concentrations). Moreover, the detergent interaction with the nanospheres has occurred in a reversible manner. This result has confirmed the higher physical stability of the investigated cyclodextrin nanocarriers in comparison to liposomes formed by phospholipids.

### 3.2. Organized Nanosystems of Amphiphilic Cyclodextrins as Non-Viral Gene Carriers

**Figure 6:**
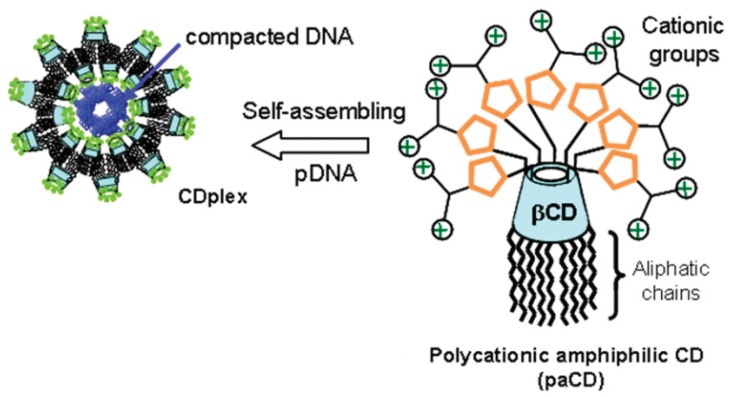
Schematic representation of polycationic amphiphilic cyclodextrins (CD) (**right**); which may form nanocomplexes (CDplexes) upon assembly with negatively charged pDNA (**left**) [80]. Reprinted with permission from [80]. Copyright (2011) American Chemical Society.

The circumstance that nanostructure-mediated non-viral nucleic-acid delivery is insufficiently developed yet for clinical applications has strongly motivated the research on novel synthetic amphiphilic cyclodextrin assemblies as potential non-viral gene carriers [43,45,46,61,62,63,64,80]. The successful entry of plasmid DNA (a multianionic macroion) into the living cells requires a complex formation between DNA and cationic carriers (CDplexes) enabling the DNA compaction and facilitating the CDplex adsorption at the negatively charged biomembranes towards cellular internalization and targeted delivery (Figure 6).

A library of polycationic amphiphilic cyclodextrins for plasmid DNA (pDNA) delivery has been investigated by O’Neill *et al.* [46,62]. The authors have shown that attachment of functional groups (relevant to development of gene carriers) has been possible on both the primary and the secondary faces of the cyclodextrin ring. In a first instance, amino groups have been conjugated on the primary-hydroxyl face and lipid chains have been anchored on the secondary-hydroxyl face of the macrocycle. Interestingly, the orientation of the lipid tails grafted on the cyclodextrin ring has been established to influence the tranfection efficiency of Caco-2 cells (human colon carcinoma), the endocytotic uptake pathway of the cyclodextrin-DNA complexes (CDplexes) in an intestinal epithelium cellular model, and their intracellular trafficking. Fluorescently labelled pDNA has been used for the cellular uptake studies of the CDplexes. It has been shown that the size and charge of the complexes have been less dominant for the cellular association of the nanocarriers as compared to their chemical structure (namely, a cationic primary face and a lipophilic secondary face) [46]. The obtained results have indicated that the cationic nanoparticulate complexes of amphiphilic cyclodextrins with pDNA have been taken by the cells through endocytosis pathways. It has been stressed that the intracellular trafficking properties of these CDplexes would require improvement for the purposes of maximal gene expression [46].

## 4. Nanosystems of Amphiphilic Cyclodextrins Mixed with Membrane-Forming Molecules and Lipids

**Figure 7:**
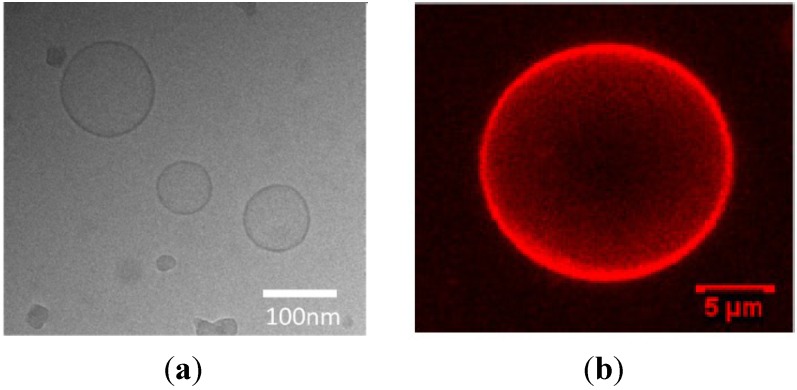
(**a**) Cryo-TEM image of binary vesicles consisting of a 1:1 mixture of an amphiphilic β-CD and lipids (DOPC/DOPE/Chol 50/25/25 mol%); (**b**) 3D reconstructed confocal laser scanning microscopy (CLSM) image of a giant unilamellar vesicle prepared from a lipid film that contains 50 mol% amphiphilic cyclodextrin (CD) (CD/DOPC/DOPE/Chol 50/25/12.5/12.5 mol%) [28]. Reprinted with permission from [28]. Copyright 2013, American Chemical Society.

The fact that modified cyclodextrins can be mixed in various proportions with phospholipids, cholesterol, and other amphiphilic molecules has been exploited for preparation of functionalized lipid membranes and organized biomimetic systems (Table 2). Hydrated mixtures of lipids and amphiphilic cyclodextrins may spontaneously assemble into unilamellar spherical vesicles (Figure 7a), giant unilamellar vesicles (Figure 7b) or multilamellar liposomes [28,49,50,51,52,53,54,55,56,57]. More sophisticated hybrid structures, involving cyclodextrin derivatives, have been prepared by controlled deposition techniques employing nanoarchitectonics means [7,14,15]. Molecular recognition reactions, together with self-assembly, have also been used for preparation of cyclodextrin-lipid bilayer architectures in nanoparticulate carriers (liposomes) [57] or in supported multilamellar membranes embedding cyclodextrin-functionalized lipid vesicles [56].

**Table 2:** Mixed systems formed by amphiphilic cyclodextrins and lipids.

Amphiphilic cyclodextrins	Co-Lipid(s)	Organized systems	Refs.
Per-(6-amino-2,3-di-*O*-hexyl) β-CD hydrochloride salt (NH_3_- β-CD-OC6)	1,2-dipalmitoyl,3-*sn*-phosphatidyl choline or 1,2-dipalmitoyl,3-*sn*-phosphatidic acid	Monolayers	[49]
Per-(6-dodecanoylamino-6-deoxy) β-CD (C_11_CONH- β-CD)	1,2-dipalmitoyl,3-phosphatidyl-choline (DPPC)	Monolayers	[49]
6^I^-(cholest-5-en-3α-ylamido)succinylamido-6^I^-deoxy-per(2,6-di-*O*-methyl) cyclomaltoheptaose (chol-DIMEB)	Dimyristoylphosphatidylcholine (DMPC)	Newtons black films	[50]
Heptakis (2,3-di-*O*-hexanoyl)-β-CD (βCD-C6)	Dimyristoylphosphatidylcholine (DMPC)	Monolayers and hydrated multibilayers	[51]
Trimethyl-α-CD-Succinyl-Cholesterol (TASC)	Dipalmitoyl-L-α-phosphatidylcholine (DPPC)	Monolayers and bilayers	[52]
Trimethyl-β-CD-Succinyl-Cholesterol (TBSC)	Dipalmitoyl-L-α-phosphatidylcholine (DPPC)	Monolayers and bilayers	[52]
Trimethyl- Β-CD-diSuccinyl-Cholesterol (TBdSC)	Dipalmitoyl-L-α-phosphatidylcholine (DPPC)	Monolayers and bilayers	[52]
Dilauryl-β-CD Dilauryl-di-2,6-*O*-methyl β-CD Dilauryl-tri-2,3,6-*O*-methyl β-CD	Dimyristoylphosphatidylcholine (DMPC)	Lamellar-phase bilayers	[53,54]
2,3-di-*O*-hexanoyl cyclomaltooctaose (γCDC6)	n-octyl-β-D-glucopyranoside	Spheres	[55]
Amphiphilic β-CD (modified on the primary face)	Dioleyl phosphatidyl ethanolamine (DOPE), dioleyl phosphatidylcholine (DOPC) and cholesterol	Three-dimensional multilayered structures	[56]
Amphiphilic β-CD (substituted with seven hydrophobic *n*-dodecyl chains on the primary face and seven hydrophilic oligo(ethylene glycol) groups on the secondary face)	Dioleyl phosphatidyl ethanolamine (DOPE), dioleyl phosphatidylcholine (DOPC) and cholesterol	Giant unilamellar vesicles (GUV), liposomes, Mixed vesicles	[28]
Mono-(N-*n*-alkyl,N,N-dimethylamino)-β-CD	Cholesterol/dipalmitoylphosphatidyl choline mixture	Three-dimensional multilayered structures of liposome type	[57]

A number of studies have focused their attention on the affinity of amphiphilic cyclodextrins for incorporation in model and biological membranes. In general, high hydrophobicity of the cyclodextrin derivatives might constitute an obstacle for their inclusion in lipid bilayer membranes owing to the preference for phase separation in two-dimensional domains. A variety of physical and physico-chemical methods (small-angle X-ray scattering (SAXS), X-ray diffraction, small-angle neutron scattering (SANS), X-ray-reflectivity, neutron reflectivity, differential scanning calorimetry (DSC), deuterium nuclear magnetic resonance (^2^H NMR), surface pressure/area isotherms, Brewster angle microscopy (BAM), atomic force microscopy (AFM), cryogenic transmission electron microscopy (cryo-TEM), and confocal laser scanning microscopy (CLSM)) have been employed to investigate these effects [11,12,13,14,15,16,17,18,19,20,21,22,23,24,25,26,27,28,29,30,31,32,33,34,35,36,37,38,39,40,41,42,43,44,45,46,47,48,49,50,51,52,53,54].

Auzély-Velty *et al.* [11] have studied the incorporation of monosubstituted cholesteryl-cyclodextrins (Chol-β-CD) into model phospholipid membranes. A lamellar phase of dimyristoyl-phosphatidylcholine (DMPC) containing varying concentrations of Chol-β-CD has been investigated by means of small-angle X-ray scattering (SAXS), differential scanning calorimetry (DSC), and ^31^P nuclear magnetic resonance (NMR). The authors have interpreted the presence of four Bragg peaks in the SAXS patterns (see Figure 2 in reference [11]) as due to a coexistence of two lamellar phases in the mixed phospholipid/amphiphilic-cyclodextrin assemblies. The peaks at 0.1005 and 0.2001 Å^−1^ have indicated the first- and second-order Bragg peak positions of the lamellar L_α_ phase of pure DMPC. The peaks at 0.0845 and 0.1672 Å^−1^ have been assigned to a coexisting lamellar phase L_CD_ (cyclodextrin-rich lamellar phase) with larger swelling. The obtained results have suggested that the Chol-β-CD molecules were entirely partitioned in the L_CD_ phase, which formed phase-separated domains from the excess L_α_-phase of pure DMPC. The microphase separation has been explained by strong attractive interactions between the hydrophobic cholesteryl-cyclodextrin molecules [11].

The mixing behavior of the amphiphilic cyclodextrin C_11_CONH-β-CD (having a hydrophilic secondary face and multiple long alkyl chains grafted on the primary face) with phospholipids has been evaluated by a thermodynamic approach [49] based on determination of monolayer surface pressure/area isotherms under dynamic conditions. Component miscibility has been assessed on the basis of the additivity rule, the Gibbs interaction free energies of mixing, and the dependence of the monolayer collapse pressure on the composition. Thermodynamically stable mixed molecular films have been obtained with slightly negative deviations from an ideal mixing behavior. This has been attributed to the hydrophobic interaction between the alkyl chains of the cyclodextrin and those of DPPC. The performed study has demonstrated the importance of the alkyl chain length (C_11_) of the cyclodextrin molecules for the mixing behavior with lipids.

The ability of amphiphilic 2,3-di-*O*-hexanoyl-β-cyclodextrin (β-CDC6), possessing multiple hydrophobic alkyl chains (C_6_) on the secondary face, to form mixed membranes with the DMPC lipid has been investigated by monolayer isotherms at the air/water interface, differential scanning calorimetry (DSC) and X-ray diffraction of hydrated membranes [51]. The β-CDC6 derivative has shown relatively weak affinity for the DMPC assemblies. Generally, insertion of guest amphiphilic cyclodextrins in lipid membranes may provoke local disturbance of the host membrane organization. The non-ideal behavior of the binary DMPC/β-CDC6 monolayers, spread at the air/water interface, has indicated the partial mixing of the components. The thermotropic properties of the hydrated phospholipid have been influenced by the presence of β-CDC6. A significant broadening of the endotherms and a depression of the chain melting temperature of DMPC has been observed. At low β-CDC6 molar fractions, a mixed lamellar phase has been evidenced in fully hydrated DMPC/β-CDC6 multibilayers (water-swollen self-assembled amphiphilic mixtures). The cyclodextrin incorporation in the DMPC lamellar phase has increased its repeat spacing at molar fractions below 7 mol%. The X-ray patterns have evidenced the formation of a new phase at a particular cyclodextrin molar content.

The insertion of monolauryl- and dilauryl-β-cyclodextrin derivatives in DMPC bilayers has been studied by deuterium NMR [53,54]. Lateral phase separation has been established between a cyclodextrin-rich phase (L_CD_) of the dilauryl derivative and a pure lipid (DMPC) phase. The L_CD_ phase has been stabilized by intermolecular hydrogen bonds between the saccharide groups at the membrane interface. The ordering of the lipid chains, induced by the monodilauryl derivative, has been analyzed as a function of the temperature-dependent phase transitions of the studied amphiphilic mixture.

**Figure 8:**
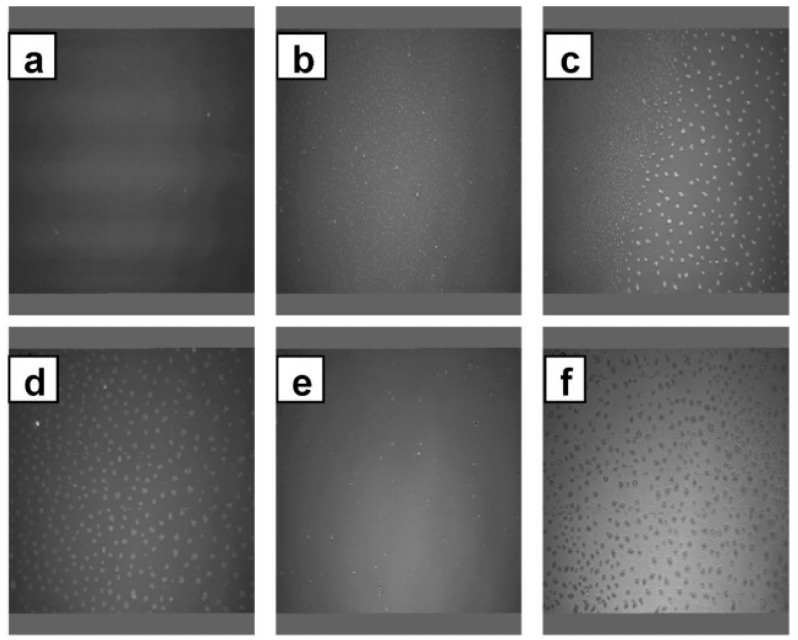
BAM micrographs (image sizes: 480 μm × 599 μm) of 33 mol% TBdSC (trimethyl-β-CD-disuccinyl-cholesterol) in DPPC Langmuir monolayers at the air/water interface at 20 °C and from upper left at (**a**) 0; (**b**) 14; (**c**) 22; (**d**) 34; (**e**) 40; and (**f**) 44 mN/m [16]. Reprinted with permission from [16]. Copyright 2011, American Chemical Society.

The in-plane organization of model membranes of DPPC and trimethyl-α-cyclodextrin-succinyl-cholesterol (TASC) or trimethyl-β-CD-disuccinyl-cholesterol (TBdSC), prepared by the Langmuir-Blodgett (LB) technique, has been characterized by Brewster angle microscopy (BAM) and atomic force microscopy (AFM) among other techniques [16,52]. The TASC derivative has been found to be miscible with the fluid DPPC monolayer phase, whereas demixing has been established at high surface pressures. The binary layers have been stable at increasing cyclodextrin percentage in the mixtures. Phase separation into domains has been better pronounced at a surface pressure of 40 mN/m. The cyclodextrins cavities in these domains have been suggested to be accessible for inclusion complexation [52]. Figure 8 shows BAM results of the miscibility of trimethyl-β-CD-disuccinyl-cholesterol (TBdSC) and DPPC in monolayers at the air/water interface [16].

**Figure 9:**
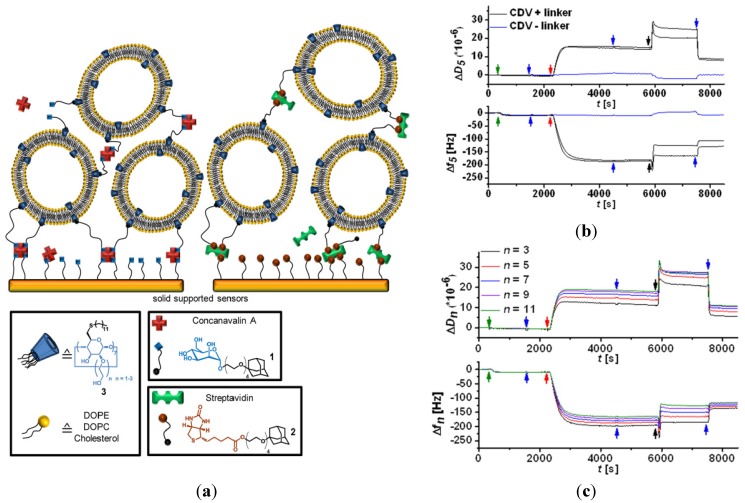
(**a**) Schematic representation of layer-by-layer deposition of vesicle multilayers mediated by noncovalent supramolecular interactions between the cyclodextrin cavities and guest units. The latter provide intervesicular links through the creation of adamantane-biotin-streptavidin or adamantane-mannose-Concavalin A complexes; (**b,c**) QCM-D data for the immobilization of cyclodextrin vesicles (CDV) via streptavidin-biotin interactions. (**b**) Fifth resonance frequency overtone of vesicle suspensions with adamantane biotin linker (black curve) and a negative control without a linker (blue curve): top, dissipation data; bottom, frequency data; (**c**) third to eleventh resonance frequency overtones of a vesicle sample with linker: (**top**), dissipation data; (**bottom**), frequency data. The arrows indicate the injection of streptavidin (green), HEPES buffer (blue), vesicles (red), and deionized water (black) [56]. Reprinted with permission from [56]. Copyright 2013, American Chemical Society.

Layer-by-layer (LbL) deposition is a key method in nanoarchitectonics [7] permitting the fabrication of functional multilayer architectures based on electrostatic and/or high affinity intermolecular interactions. A hierarchical supramolecular nanoarchitecture, involving cyclodextrin-containing vesicles, has been prepared by a biomimetic approach [56]. Three-dimensional vesicle multilayers (mimicking cellular assembly constructs) have been produced in a layer-by-layer fashion using noncovalent interactions (cyclodextrin-adamantane, biotin-streptavidin and mannose-Concavalin A) allowing the formation of intervesicular bridges (Figure 9). Bifunctional ligand molecules (adamantane-biotin and adamantane-mannose) have been chosen for the formation of inclusion complexes with the cyclodextrin cavities embedded in the vesicles (on one hand) and with the protein functionalities serving as intervesicular linkers (on the other hand). Repeating deposition cycles, exploiting host-guest chemistry and affinity interactions, have yielded multilayers of immobilized vesicles on sensor surfaces.

“Bottom-up” assembly of binary phospholipid/cholesterol liposomes with amphiphilic cyclodextrin nanocavities (synthetic mono-(*N*-*n*-alkyl,*N,N-*dimethylamino)-β-cyclodextrin, DMA-C_n_-CD) and subsequent molecular recognition reaction of the nanocavities with adamantoylglucose ligands has permitted the design of supramolecular constructs for targeting of the blood-brain-barrier (BBB) [57]. The obtained liposome nanoparticles have been decorated by glucose ligands that have been surface-exposed to bind the receptors at the BBB. The suggested supramolecular strategy has aimed to evaluate the impact of saccharide ligands on the transport of cyclodextrin-modified liposomes through the BBB. Indeed, the glucose ligand-functionalized “supramolecular” liposomes have shown a 5-fold increased capacity to enter the BBB-endothelial cells as compared to nonfunctionalized naked liposomes. Fluorescence measurements after 4 h of liposome incubation have established that 30% of the saccharide-coated liposomes have been incorporated into the endothelial cells due to the recognition process by the BBB receptors that have provoked enhanced interactions. At the same time, only 6% of the naked liposomes (lacking surface decoration by glucose-cyclodextrin complexes) have been able to permeate the BBB cellular model [57]. These results have elucidated the mechanism of nanocarrier transport through the BBB model suggesting that the liposomes may reach the cytoplasm through a non-specific pathway.

## 5. Potential Application of Supramolecular Assemblies of Amphiphilic Cyclodextrins

Nanoassemblies of fluorinated amphiphilic cyclodextrins have been used as drug carriers of a novel indenoindole-type inhibitor of the enzyme casein kinase 2 (CK2) upon tumour targeting [41]. The purpose of the study has been to improve the drug (1-amino-5-isopropyl-5,6,7,8-tetrahydroindeno[1,2-*b*]indole-9,10-dione) biodisponibility through nanoencapsulation and inclusion complexation. Nanoparticulate complexes between the indenoindole CK2 inhibitor and different amphiphilic cyclodextrin derivatives have been produced through nanoprecipitation. The encapsulation efficiency of the fluorinated amphiphilic cyclodextrin hexakis[6-deoxy-6-(3-perfluorohexyl propanethio)-2,3-di-*O*-methyl]-α-cyclodextrin (α-C6F13) nanocarriers for the indenoindole drug has been determined to be 65%, which corresponded to a considerable advance in pharmaceutical nanotechnology. The indenoindole CK2 inhibitor-loaded cyclodextrin nanospheres have shown sustained release profiles over hours. The results obtained with diverse cancer cell lines (e.g., LNCaP, PC-3, MCF-7, Caco-2) have appeared to be promising for future *in vivo* applications of the fluorinated cyclodextrin delivery systems in targeting of breast, liver, pancreas, and prostate cancer using new protein kinase CK2 inhibitors.

Emulsion-solvent evaporation [58] and nanoprecipitation techniques [59] have been employed for preparation of anticancer drug delivery systems of improved safety thanks to the use of amphiphilic cyclodextrin derivatives (SC16OH and 6-*O*-CAPRO-β-CD) (Table 3). The encapsulation efficiency of docetaxel in SC16OH nanocarriers has been determined to be 100%. These carriers have displayed vesicular shapes and a mean particle diameter of about 95 nm. An important characteristic has been the established sustained release of the drug over 8 weeks [58]. Therefore, the amphiphilic SC16OH cyclodextrin nanocarriers have shown essential promise for solid tumour therapy.

**Table 3:** Amphiphilic cyclodextrins derivatives and their potential application fields.

Amphiphilic CDs	Systems	Potential applications	Refs.
Heptakis (2-*O*-oligo(ethyleneoxide)-6-hexadecylthio-)-β-CD (SC16OH)	Nanoparticles	Drug carriers (docetaxel)	[58]
6-*O*-CAPRO-β-CD	Nanoparticles (nanospheres, nanocapsules, vesicles)	Drug carriers (paclitaxel, camptothecin)	[59,60]
βCD-C6/6-*N*-CAPRO-β-CD 6-*N*-MYRISTO-β-CD	Nanospheres Nanocapsules	Drug carriers (progesterone, camptothecin)	[35,36,60]
Oligoethyleneimine-βCD	CDplexes	Gene delivery	[61]
Heptakis[6-(2-amino-ethylthio)-6-deoxy-2-*O*-octylsulfanylpropyl]-β-cyclodextrin hepta-N-trifluoroacetate	CDplexes	Gene delivery	[62]
Heptakis[6-diBoc-guanidinoethylthio-2-*O-*(N-(ω-(p-methoxybenzamido)-PEG440-yl)-1′H-triazole-4’-yl-methyl)]-β-cyclodextrin	CDplexes	Gene delivery	[63]
Heptakis[6-deoxy-6-(2-(N’-(2-(N,N-di-(2-aminoethyl)amino)ethyl)thioureido)ethylthio)-2,3-di-*O*-hexanoyl] cyclomaltoheptaose	CDplexes	Gene delivery	[64]
Heptakis(6-dodecylthio-2-oligo(ethylenoxide)-β-cyclodextrin-2-(4-(phenyldiazenyl)phenoxy) acetate	Vesicles	Phototherapy	[65]
Heptakis(2-*O*-oligo(ethylene oxide)-6-hexylthio)-β-CD (SC6OH)	Nanoparticles	Phototherapy	[30]
SC_6_NH_3_-β-CD; SC_16_NH_3_-β-CD	Multilayer films	Photoresponsive multilayer films	[66]
Peptidyl-β-cyclodextrins	Assemblies with biological membranes	Artificial receptors	[67]
Amphiphilic β-cyclodextrins with *n*-dodecyl groups and *n-*hexadecyl chains on both faces	Assemblies with biological membranes	Artificial receptors	[28]

The investigation of Bilensoy *et al.* [59] has been motivated by the problems of recrystallization and precipitation of the paclitaxel drug after injection from a commercial formulation. (The associated undesired effects have caused severe necrosis in cancer patients). The new cyclodextrin nanoparticles [59], encapsulating paclitaxel, have been characterized by spherical shapes, high drug encapsulation rate (65%), and good physical stability in the absence of surfactants. They have caused essentially lower hemolysis of erythrocytes in comparison to the commercial drug formulation (cremophor/ethanol 50/50 v/v). The cytotoxicity of the blank cyclodextrin (6-*O*-CAPRO-β-CD) nanocarriers to L929 cells has also been lower with respect to the cremophor/ethanol commercial formulation. The anticancer efficacy of paclitaxel-loaded cyclodextrin nanoparticles has been evaluated in a MCF-7 cell line. The results have been equivalent to those with the commercial vehicles [59].

Cyclodextrin-based nanoparticles, produced by self-assembly of amphiphilic polycationic derivatives (with hydrophobic chains grafted on the primary face and cationic groups conjugated to the secondary face of the ring or vice versa), have been investigated as carriers of siRNA (short interfering RNAs) in therapeutic approaches requiring gene silencing [43,44,48,63]. Godinho *et al.* [44] have discussed the advantages of chemically-modified β-cyclodextrin (SC12 CD) particles, characterized by low toxicity and low immunogenicity, for siRNA delivery to the central nervous system. Cyclodextrin-siRNA nanoparticulate complexes (~200 nm in diameter) have been assembled as stable entities in artificial cerebrospinal fluid. Towards transfection purposes, they have been injected into the striatum of R6/2 mice in an *in vitro* model of Huntington’s disease (HD). The transfected nanoassemblies have been able to significantly reduce the expression of the gene of the toxic Huntingtin (HTT) protein (by ~85% after 4 h) [44]. Furthermore, cyclodextrin-based siRNA carriers have been suggested to be applicable also to other neurodegenerative diseases, such as Alzheimer’s, Parkinson, and amyotrophic lateral sclerosis (ALS).

Functionalization of amphiphilic cyclodextrin molecules by receptor ligands has been employed in targeted gene delivery [43,45]. For instance, CDplexes have been obtained by self-assembly of polycationic amphiphilic cyclodextrin (containing 14 hexanoyl chains on the secondary face and 14 primary amino groups at the primary face) and plasmid DNA (pDNA). The surface of the CDplex nanoparticles has been decorated by folic acid [43]. Folic acid ligands have been chosen because the folate receptor is highly over-expressed in various carcinomas. Thus, the enhanced transfection of the CDplexes (structural organization shown in Figure 2, bottom right panel) in human cervix adenocarcinoma HeLa cells has been favored by the folate-receptor-mediated internalization of the folate-decorated nanocarriers.

Table 3 presents valuable examples of recently reported applications of organized amphiphilic cyclodextrin assemblies [28,30,35,36,58,59,60,61,62,63,64,65,66,67]. For a broader range of potential applications of cyclodextrin-based nanomaterials, the reader is advised to consult previous publications [68,69,70,71,72,73,74,75,76,77,78,79,80,81,82].

## 6. Conclusions

Nanoarchitectonics has emerged as a fast developing field [7], which has recently encompassed new directions of research on cationic and amphiphilic cyclodextrins, *i.e.*, nanoarchitectonics with cavity-type self-assembled nanomaterials. The modulation of the hydrophilic–hydrophobic balance of the modified cyclodextrin molecules governs the type of the self-assembled nanostructures and their complexes with guest molecules. This causes corresponding differences in the interaction of the amphiphilic nanoassemblies with living cells and tissues, as well as changes in their capacity to encapsulate and deliver active ingredients. One of the interesting applications of the innovative cyclodextrin-based supramolecular complexes includes the neuronal siRNA delivery. The projected structural modifications at both faces of the cyclodextrin macrocycles (e.g., lipophilic groups on the secondary face and cationic groups on the primary face) are responsible for the control of the transfection efficiency of the siRNA nanocarriers. The siRNA nanocarrier ability to mediate gene silencing is dependent on the relative facial positioning of the lipophilic and the cationic moieties in the amphiphilic cyclodextrin structure (primary or secondary sides of the molecule). It is noteworthy that the cyclodextrin-siRNA complexes were able to provide protection of the siRNAs from serum nucleases. In addition, site-specific gene delivery using polycationic amphiphilic cyclodextrins has been suggested as a safety method for neurodegenerative disease therapies. Oral delivery of gene therapeutics through complexation with cationic amphiphilic cyclodextrin carriers should avoid invasive surgery and may be recommended for treatment of colon cancer and inflammatory bowel disease.

Further possibilities exist for the use of amphiphilic cyclodextrins in drug delivery applications. Photosensitizer drugs, encapsulated in amphiphilic cyclodextrin particles, have efficiently induced photodynamic damage of cancer cells. Advancements have been achieved also in treatment of solid tumours by docetaxel and paclitaxel using novel cyclodextrin-based nanocarriers obtained by emulsion-solvent evaporation or nanoprecipitation methods. They have been characterized by high drug encapsulation efficiency (65% for paclitaxel and 100% for docetaxel). Sustained drug release provided up to two months, as well as lower toxicity and lack of hemolysis, has presented evidence for the beneficial properties of the amphiphilic cyclodextrin nanocarriers with regards to commercially available antitumor drug formulations. Affinity of the modified cyclodextrins to biological membranes has been revealed by studies of mixed phospholipid/cyclodextrin assemblies.
